# Supplementary material for: Hot spot prediction in protein-protein interactions by an ensemble system
Source: BMC Syst Biol. 2018 Dec 31;12(Suppl 9):132. doi: 10.1186/s12918-018-0665-8 (PMC6311905; doi:10.1186/s12918-018-0665-8)
Supplement: Supplementary file 2 — All relevant MATLAB code. A simple Matlab implement of our predictor. (ZIP 19,742 kb) [file 12918_2018_665_MOESM2_ESM.zip › Additional file 2/Read ASA/ASA website/dbMPIKT/3SE9L.html]

 NetSurfP - Protein Surface Accessibility and Secondary Structure Predictions

  

|  |  |  |
| --- | --- | --- |
|  |  | NetSurfP - Protein Surface Accessibility and Secondary Structure Predictions  Technical University of Denmark |

  


---

```
# For publication of results, please cite:
# A generic method for assignment of reliability scores applied to solvent accessibility predictions.
# Bent Petersen, Thomas Nordahl Petersen, Pernille Andersen, Morten Nielsen and Claus Lundegaard
# BMC Structural Biology 2009, 9:51 doi:10.1186/1472-6807-9-51
#
# Column 1: Class assignment - B for buried or E for Exposed - Threshold: 25% exposure, but not based on RSA
# Column 2: Amino acid
# Column 3: Sequence name
# Column 4: Amino acid number
# Column 5: Relative Surface Accessibility - RSA
# Column 6: Absolute Surface Accessibility
# Column 7: Z-fit score for RSA prediction
# Column 8: Probability for Alpha-Helix
# Column 9: Probability for Beta-strand
# Column 10: Probability for Coil
E E  3SE9L                  1    0.808 141.140   0.694   0.003   0.003   0.994
B I  3SE9L                  2    0.268  49.561  -0.952   0.004   0.616   0.381
E V  3SE9L                  3    0.436  67.029   1.203   0.001   0.900   0.099
B L  3SE9L                  4    0.129  23.602   0.274   0.001   0.900   0.099
E T  3SE9L                  5    0.438  60.806   0.355   0.002   0.816   0.182
B Q  3SE9L                  6    0.155  27.647   0.577   0.004   0.514   0.481
E S  3SE9L                  7    0.371  43.469   0.132   0.004   0.138   0.858
E P  3SE9L                  8    0.498  70.638  -1.154   0.018   0.019   0.964
E G  3SE9L                  9    0.551  43.332  -1.188   0.018   0.088   0.893
E T  3SE9L                 10    0.390  54.162  -1.521   0.021   0.451   0.528
B L  3SE9L                 11    0.255  46.690  -0.319   0.021   0.756   0.223
E S  3SE9L                 12    0.505  59.163  -0.642   0.018   0.846   0.136
B L  3SE9L                 13    0.216  39.531   0.076   0.021   0.756   0.223
E S  3SE9L                 14    0.442  51.849   0.305   0.004   0.420   0.576
E P  3SE9L                 15    0.519  73.646  -0.133   0.004   0.085   0.910
E G  3SE9L                 16    0.724  56.995  -0.950   0.005   0.015   0.979
E E  3SE9L                 17    0.465  81.270   0.565   0.004   0.138   0.858
E T  3SE9L                 18    0.522  72.388   0.975   0.003   0.718   0.279
B A  3SE9L                 19    0.063   6.954   0.110   0.001   0.900   0.099
E S  3SE9L                 20    0.419  49.072   1.638   0.001   0.959   0.040
B L  3SE9L                 21    0.054   9.869   0.363   0.001   0.959   0.040
E S  3SE9L                 22    0.338  39.649   0.910   0.001   0.959   0.040
B C  3SE9L                 23    0.030   4.198  -0.122   0.001   0.900   0.099
E T  3SE9L                 24    0.395  54.773   0.747   0.001   0.900   0.099
B A  3SE9L                 25    0.181  19.957  -1.134   0.002   0.816   0.182
E A  3SE9L                 26    0.331  36.520  -0.762   0.005   0.336   0.660
E S  3SE9L                 27    0.609  71.375  -2.357   0.005   0.045   0.951
E Y  3SE9L                 28    0.442  94.520  -1.875   0.005   0.045   0.951
B G  3SE9L                 29    0.255  20.045  -0.868   0.019   0.141   0.840
B H  3SE9L                 30    0.211  38.472  -0.082   0.004   0.514   0.481
B M  3SE9L                 31    0.031   6.283  -0.131   0.018   0.846   0.136
B T  3SE9L                 32    0.118  16.367   0.097   0.018   0.846   0.136
B W  3SE9L                 33    0.033   7.912  -0.058   0.018   0.846   0.136
B Y  3SE9L                 34    0.066  14.083   0.195   0.011   0.918   0.071
B Q  3SE9L                 35    0.163  29.112   0.240   0.018   0.846   0.136
B K  3SE9L                 36    0.179  36.779   0.410   0.004   0.616   0.381
E K  3SE9L                 37    0.431  88.616   1.488   0.004   0.138   0.858
E P  3SE9L                 38    0.540  76.555   0.043   0.005   0.015   0.979
E G  3SE9L                 39    0.814  64.085  -2.157   0.003   0.003   0.994
E Q  3SE9L                 40    0.456  81.406   0.420   0.018   0.019   0.964
E P  3SE9L                 41    0.394  55.852  -0.074   0.018   0.047   0.935
B P  3SE9L                 42    0.088  12.459   0.327   0.021   0.279   0.699
E K  3SE9L                 43    0.312  64.281   0.614   0.003   0.718   0.279
B L  3SE9L                 44    0.127  23.272   0.268   0.001   0.900   0.099
B L  3SE9L                 45    0.050   9.063   0.179   0.001   0.959   0.040
B I  3SE9L                 46    0.028   5.254  -0.285   0.001   0.959   0.040
B F  3SE9L                 47    0.101  20.210  -0.331   0.001   0.959   0.040
B A  3SE9L                 48    0.111  12.188  -1.003   0.001   0.900   0.099
B T  3SE9L                 49    0.118  16.325  -1.255   0.004   0.616   0.381
B S  3SE9L                 50    0.201  23.557  -1.057   0.022   0.359   0.619
E K  3SE9L                 51    0.521 107.149  -1.276   0.019   0.141   0.840
E R  3SE9L                 52    0.415  94.966  -0.846   0.018   0.047   0.935
E A  3SE9L                 53    0.553  60.919  -2.182   0.018   0.047   0.935
E S  3SE9L                 54    0.680  79.661  -1.384   0.018   0.047   0.935
E G  3SE9L                 55    0.524  41.239  -1.330   0.018   0.047   0.935
E I  3SE9L                 56    0.413  76.479  -0.581   0.018   0.019   0.964
E P  3SE9L                 57    0.572  81.110  -1.283   0.005   0.015   0.979
E D  3SE9L                 58    0.491  70.695  -1.733   0.004   0.085   0.910
E R  3SE9L                 59    0.296  67.899  -0.491   0.004   0.420   0.576
B F  3SE9L                 60    0.083  16.578  -1.235   0.003   0.718   0.279
B S  3SE9L                 61    0.235  27.530  -1.241   0.004   0.616   0.381
B G  3SE9L                 62    0.182  14.316  -2.962   0.023   0.655   0.322
E S  3SE9L                 63    0.404  47.384  -1.102   0.004   0.514   0.481
E Q  3SE9L                 64    0.446  79.691  -1.321   0.004   0.420   0.576
E F  3SE9L                 65    0.484  97.159  -2.108   0.004   0.197   0.799
E G  3SE9L                 66    0.623  48.999  -2.216   0.005   0.045   0.951
E K  3SE9L                 67    0.483  99.353  -1.503   0.004   0.138   0.858
E Q  3SE9L                 68    0.413  73.762  -0.115   0.004   0.514   0.481
B Y  3SE9L                 69    0.106  22.716  -0.439   0.002   0.816   0.182
B T  3SE9L                 70    0.199  27.587  -0.244   0.002   0.816   0.182
B L  3SE9L                 71    0.058  10.565   0.054   0.001   0.900   0.099
B T  3SE9L                 72    0.246  34.148  -0.357   0.001   0.900   0.099
B I  3SE9L                 73    0.055  10.119   0.176   0.002   0.816   0.182
E T  3SE9L                 74    0.317  43.954  -0.031   0.018   0.846   0.136
E R  3SE9L                 75    0.350  80.058  -0.605   0.023   0.655   0.322
B M  3SE9L                 76    0.257  51.526  -0.449   0.022   0.359   0.619
E E  3SE9L                 77    0.416  72.640   0.129   0.020   0.205   0.775
E P  3SE9L                 78    0.402  57.044  -0.847   0.056   0.142   0.802
E E  3SE9L                 79    0.608 106.235  -1.836   0.052   0.084   0.864
E D  3SE9L                 80    0.545  78.535  -1.136   0.053   0.043   0.903
E F  3SE9L                 81    0.410  82.307  -1.168   0.118   0.150   0.732
E A  3SE9L                 82    0.356  39.176  -1.139   0.066   0.296   0.638
E R  3SE9L                 83    0.385  88.234   0.176   0.069   0.386   0.545
B Y  3SE9L                 84    0.174  37.227   0.362   0.074   0.484   0.442
B Y  3SE9L                 85    0.187  39.941   0.025   0.021   0.451   0.528
E C  3SE9L                 86    0.354  49.702  -1.007   0.022   0.359   0.619
B Q  3SE9L                 87    0.257  45.864  -0.892   0.022   0.359   0.619
B Q  3SE9L                 88    0.274  48.847  -0.753   0.021   0.279   0.699
E L  3SE9L                 89    0.354  64.781  -1.236   0.064   0.216   0.721
E E  3SE9L                 90    0.424  74.143  -1.625   0.064   0.216   0.721
E F  3SE9L                 91    0.441  88.448  -1.649   0.118   0.150   0.732
E F  3SE9L                 92    0.379  76.005  -1.955   0.113   0.043   0.844
E G  3SE9L                 93    0.411  32.354  -2.217   0.058   0.017   0.925
E Q  3SE9L                 94    0.389  69.493  -1.484   0.113   0.043   0.844
E G  3SE9L                 95    0.364  28.607  -0.958   0.113   0.087   0.800
E T  3SE9L                 96    0.289  40.112  -1.145   0.113   0.087   0.800
B R  3SE9L                 97    0.227  52.075  -1.556   0.118   0.150   0.732
B L  3SE9L                 98    0.287  52.531  -1.338   0.216   0.235   0.548
E E  3SE9L                 99    0.400  69.863  -1.293   0.216   0.235   0.548
E I  3SE9L                100    0.392  72.575  -1.265   0.125   0.227   0.648
E R  3SE9L                101    0.449 102.707  -1.521   0.056   0.142   0.802
E R  3SE9L                102    0.398  91.096  -1.315   0.056   0.142   0.802
E T  3SE9L                103    0.402  55.813  -1.564   0.019   0.141   0.840
E V  3SE9L                104    0.423  65.000  -1.639   0.019   0.141   0.840
E A  3SE9L                105    0.435  47.915  -1.735   0.019   0.141   0.840
E A  3SE9L                106    0.485  53.425  -1.516   0.018   0.088   0.893
E P  3SE9L                107    0.405  57.455  -1.535   0.018   0.088   0.893
E S  3SE9L                108    0.464  54.404  -1.311   0.020   0.205   0.775
E V  3SE9L                109    0.370  56.884  -1.588   0.021   0.279   0.699
E F  3SE9L                110    0.450  90.295  -1.718   0.021   0.279   0.699
E I  3SE9L                111    0.562 103.896  -2.443   0.021   0.279   0.699
E F  3SE9L                112    0.343  68.900  -2.103   0.019   0.141   0.840
E P  3SE9L                113    0.396  56.136  -1.844   0.018   0.047   0.935
B P  3SE9L                114    0.251  35.560  -2.806   0.018   0.019   0.964
B S  3SE9L                115    0.276  32.324  -2.035   0.018   0.047   0.935
E D  3SE9L                116    0.412  59.441  -1.934   0.018   0.088   0.893
E E  3SE9L                117    0.502  87.769  -2.036   0.019   0.141   0.840
E Q  3SE9L                118    0.334  59.617  -2.442   0.018   0.088   0.893
B L  3SE9L                119    0.279  51.158  -1.158   0.018   0.047   0.935
E K  3SE9L                120    0.550 113.073  -0.806   0.018   0.019   0.964
E S  3SE9L                121    0.769  90.139  -0.829   0.005   0.015   0.979
E G  3SE9L                122    0.582  45.788  -1.392   0.005   0.015   0.979
E T  3SE9L                123    0.474  65.744   0.328   0.005   0.336   0.660
B A  3SE9L                124    0.098  10.756  -0.102   0.002   0.816   0.182
B S  3SE9L                125    0.240  28.105   0.879   0.001   0.900   0.099
B V  3SE9L                126    0.042   6.517   0.120   0.001   0.959   0.040
B V  3SE9L                127    0.042   6.409  -0.602   0.001   0.959   0.040
B C  3SE9L                128    0.017   2.345   1.253   0.001   0.959   0.040
B L  3SE9L                129    0.101  18.548   0.530   0.001   0.959   0.040
B L  3SE9L                130    0.024   4.413   0.289   0.001   0.900   0.099
B N  3SE9L                131    0.207  30.378   0.521   0.002   0.816   0.182
B N  3SE9L                132    0.297  43.466  -1.154   0.004   0.514   0.481
B F  3SE9L                133    0.339  67.957  -1.848   0.005   0.262   0.733
B Y  3SE9L                134    0.267  57.079  -0.455   0.004   0.138   0.858
E P  3SE9L                135    0.415  58.818  -1.580   0.004   0.085   0.910
E R  3SE9L                136    0.649 148.529  -2.292   0.004   0.085   0.910
E E  3SE9L                137    0.372  65.023  -0.802   0.005   0.336   0.660
B A  3SE9L                138    0.079   8.695  -0.132   0.003   0.718   0.279
B K  3SE9L                139    0.252  51.734   0.125   0.001   0.900   0.099
B V  3SE9L                140    0.043   6.594   0.182   0.001   0.900   0.099
B Q  3SE9L                141    0.191  34.148   0.542   0.011   0.918   0.071
B W  3SE9L                142    0.044  10.654   0.095   0.011   0.918   0.071
E K  3SE9L                143    0.284  58.481   0.803   0.001   0.900   0.099
E V  3SE9L                144    0.342  52.581   0.696   0.004   0.616   0.381
E D  3SE9L                145    0.637  91.763  -0.002   0.005   0.045   0.951
E N  3SE9L                146    0.693 101.470  -1.432   0.005   0.015   0.979
E A  3SE9L                147    0.679  74.848  -0.832   0.004   0.138   0.858
E L  3SE9L                148    0.597 109.366  -1.505   0.004   0.197   0.799
E Q  3SE9L                149    0.437  77.995  -0.523   0.004   0.197   0.799
E S  3SE9L                150    0.524  61.378  -1.397   0.004   0.197   0.799
E G  3SE9L                151    0.460  36.194  -1.643   0.004   0.197   0.799
E N  3SE9L                152    0.402  58.824  -1.889   0.004   0.420   0.576
B S  3SE9L                153    0.274  32.101  -1.019   0.003   0.718   0.279
B Q  3SE9L                154    0.291  51.919  -1.294   0.002   0.816   0.182
E E  3SE9L                155    0.436  76.239  -1.062   0.021   0.756   0.223
E S  3SE9L                156    0.305  35.758  -1.731   0.021   0.451   0.528
E V  3SE9L                157    0.351  53.979  -0.533   0.022   0.359   0.619
E T  3SE9L                158    0.449  62.290  -0.959   0.022   0.359   0.619
E E  3SE9L                159    0.392  68.500  -0.981   0.021   0.279   0.699
E Q  3SE9L                160    0.486  86.746  -1.078   0.021   0.279   0.699
E D  3SE9L                161    0.598  86.172  -2.061   0.020   0.205   0.775
E S  3SE9L                162    0.613  71.902  -1.766   0.004   0.085   0.910
E K  3SE9L                163    0.484  99.518  -1.106   0.005   0.045   0.951
E D  3SE9L                164    0.725 104.501  -1.335   0.005   0.015   0.979
E S  3SE9L                165    0.456  53.455  -1.579   0.005   0.045   0.951
E T  3SE9L                166    0.324  44.883   0.261   0.004   0.514   0.481
B Y  3SE9L                167    0.077  16.434  -1.493   0.003   0.718   0.279
B S  3SE9L                168    0.102  11.966  -1.133   0.001   0.900   0.099
B L  3SE9L                169    0.070  12.817  -1.188   0.001   0.900   0.099
B S  3SE9L                170    0.138  16.185  -0.344   0.001   0.959   0.040
B S  3SE9L                171    0.041   4.829  -1.185   0.001   0.959   0.040
B T  3SE9L                172    0.179  24.855   0.200   0.001   0.959   0.040
B L  3SE9L                173    0.040   7.416   0.102   0.001   0.900   0.099
E T  3SE9L                174    0.298  41.374   0.695   0.001   0.900   0.099
B L  3SE9L                175    0.048   8.880   0.554   0.002   0.816   0.182
E S  3SE9L                176    0.380  44.489   1.486   0.004   0.420   0.576
E K  3SE9L                177    0.434  89.233  -0.584   0.020   0.205   0.775
E A  3SE9L                178    0.569  62.704  -0.946   0.019   0.141   0.840
E D  3SE9L                179    0.541  77.929   0.842   0.020   0.205   0.775
B Y  3SE9L                180    0.169  36.137  -1.288   0.019   0.141   0.840
E E  3SE9L                181    0.681 118.883   0.302   0.005   0.045   0.951
E K  3SE9L                182    0.641 131.833   0.539   0.005   0.045   0.951
E H  3SE9L                183    0.634 115.306  -0.415   0.005   0.045   0.951
E K  3SE9L                184    0.523 107.622   0.351   0.005   0.336   0.660
E V  3SE9L                185    0.430  66.137   1.652   0.002   0.816   0.182
B Y  3SE9L                186    0.068  14.467   0.831   0.001   0.959   0.040
E A  3SE9L                187    0.312  34.338   1.740   0.001   0.959   0.040
B C  3SE9L                188    0.025   3.524   0.701   0.001   0.959   0.040
E E  3SE9L                189    0.284  49.545   1.443   0.001   0.959   0.040
B V  3SE9L                190    0.077  11.820  -0.414   0.001   0.900   0.099
E T  3SE9L                191    0.433  60.015   0.598   0.001   0.959   0.040
B H  3SE9L                192    0.146  26.521   0.366   0.002   0.816   0.182
E Q  3SE9L                193    0.664 118.662   0.100   0.005   0.262   0.733
E G  3SE9L                194    0.640  50.368  -1.794   0.005   0.045   0.951
E L  3SE9L                195    0.394  72.105  -0.726   0.005   0.045   0.951
E S  3SE9L                196    0.658  77.106  -0.235   0.018   0.047   0.935
E S  3SE9L                197    0.539  63.136   0.141   0.019   0.141   0.840
E P  3SE9L                198    0.457  64.905   0.235   0.022   0.359   0.619
B V  3SE9L                199    0.179  27.528  -0.180   0.003   0.718   0.279
E T  3SE9L                200    0.543  75.342   0.505   0.001   0.900   0.099
E K  3SE9L                201    0.292  60.064   1.375   0.001   0.900   0.099
E S  3SE9L                202    0.450  52.799   0.089   0.001   0.900   0.099
B F  3SE9L                203    0.099  19.809   0.940   0.001   0.900   0.099
E N  3SE9L                204    0.510  74.693   1.517   0.004   0.616   0.381
E R  3SE9L                205    0.462 105.752   0.521   0.020   0.205   0.775
E G  3SE9L                206    0.627  49.337  -1.388   0.018   0.088   0.893
E E  3SE9L                207    0.658 114.953   1.531   0.018   0.088   0.893
E C  3SE9L                208    0.518  72.783  -1.331   0.003   0.003   0.994

Explain the output.  Go back.
```

---
